# Understanding global variation in breast cancer mortality

**DOI:** 10.1093/bjr/tqaf148

**Published:** 2025-07-03

**Authors:** Marwa M A Elbasheer, David Dodwell, Toral Gathani

**Affiliations:** Cancer Epidemiology Unit, Nuffield Department of Population Health, University of Oxford, Oxford, OX3 7LF, United Kingdom; Nuffield Department of Population Health, University of Oxford, Oxford, OX3 7LF, United Kingdom; Oxford University Hospitals NHS Foundation Trust, Oxford, OX3 7LE, United Kingdom; Cancer Epidemiology Unit, Nuffield Department of Population Health, University of Oxford, Oxford, OX3 7LF, United Kingdom; Oxford University Hospitals NHS Foundation Trust, Oxford, OX3 7LE, United Kingdom

**Keywords:** breast cancer, mortality, global health, conflict, cancer care

## Abstract

Breast cancer is an important global health challenge. Among women, it is the most commonly diagnosed cancer and the leading cause of cancer-related mortality. There are significant variations in the incidence and mortality rates globally with highest incidence and lowest mortality observed in more developed countries. The number of women diagnosed with breast cancer is projected to rise to ∼3 million annual cases by 2050 from ∼2 million in 2022, and the number of breast cancer-related deaths is projected to rise to over 1 million by 2050 from ∼660 000 in 2022. The largest increases in both incidence and mortality will be in less developed regions of the world. Over the last 40 years, significant gains have been made in reducing breast cancer mortality in more developed countries, but significant challenges remain in tackling the less favourable mortality rates in less developed countries. In this article, we discuss the global variation in breast cancer mortality rates. We used Sudan to present a case study of the devastating impact of conflict on cancer care.

## Introduction

Breast cancer is an important global health challenge. Among women, breast cancer is the most commonly diagnosed cancer, with an estimated annual 2.3 million incident cases (representing 1 in 4 cancers diagnosed), and the leading cause of cancer-related mortality with over 660 000 cancer-related deaths (representing 1 in 7 deaths).^1^

Estimates suggest that by 2050, over 3 million women will be diagnosed with breast cancer annually, and there will be over a million breast cancer-related deaths, with the largest increase expected to occur in low- and middle-income countries.^2^

A significant reduction in breast cancer mortality rates in more developed countries has been observed over the last 40 years largely attributed to a combination of earlier detection and diagnosis achieved through population-based screening and more streamlined referral pathways between primary and secondary care, combined with the availability of more effective treatments. In comparison, less favourable outcomes from breast cancer continue to be observed in countries with fewer resources.

In this article, we discuss the key drivers that have led to reductions in breast cancer mortality in more developed countries and the challenges that remain for implementing high-quality breast cancer care in poorer regions. We will use Sudan as a case study of what impact conflict has on cancer care.

## Global variation in breast cancer mortality rates

Breast cancer is the leading cause of cancer-related death in women in 112 countries. In several developed countries, lung cancer is the leading cause of cancer-related deaths as a reflection of the increased use of tobacco among females in the latter half of the last century, and cervical cancer remains the leading cause of cancer-related death in women in large parts of sub-Saharan Africa and parts of Asia.^1^

Breast cancer incidence and mortality rates are highly correlated with development with the highest incidence and lowest mortality rates observed in those countries with the highest levels of development.

Variations in breast cancer incidence are largely explained by differences in population demographics and variations in known lifestyle and reproductive risk factors for the disease.^3^ There are also significant variations in the distribution of average age at presentation, with roughly 50% of breast cancers occurring in women aged <50 years in less developed countries, compared to 20% in more developed countries.^4^

**Figure 1.**
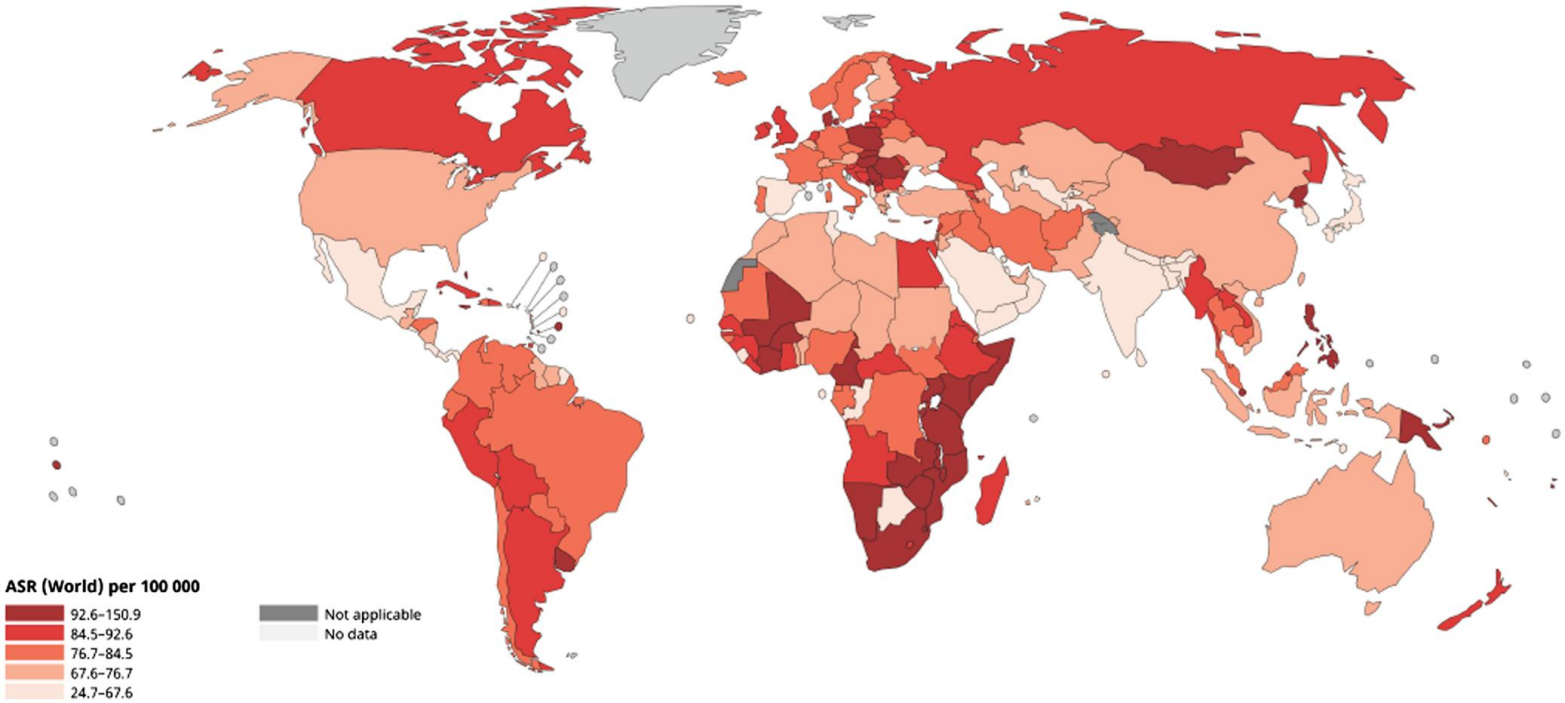
Age standardized rate (world) per 100 000 for breast cancer mortality in women in 2022. © International Agency for Research on https://gco.iarc.who.int/today. Data version: Globocan 2022 (version 1.1)—February 08, 2024. Downloaded March 2025.

The highest mortality rates for breast cancer are observed in less developed regions of the world (Figure 1). The mortality incidence ratio can be used as a measure of relative survival in populations and ranges from a favourable 18% in the most developed countries to 56% in less developed countries.^2^ For less developed regions of the world, where the average age of breast cancer presentation is younger and mortality rates are higher, the unfavourable mortality rates result in a significant societal impact. In 2020, it was estimated that 50% of maternal orphans are a result of breast cancer, and the vast majority of these live in Asia and Africa.^5^

**Figure 2.**
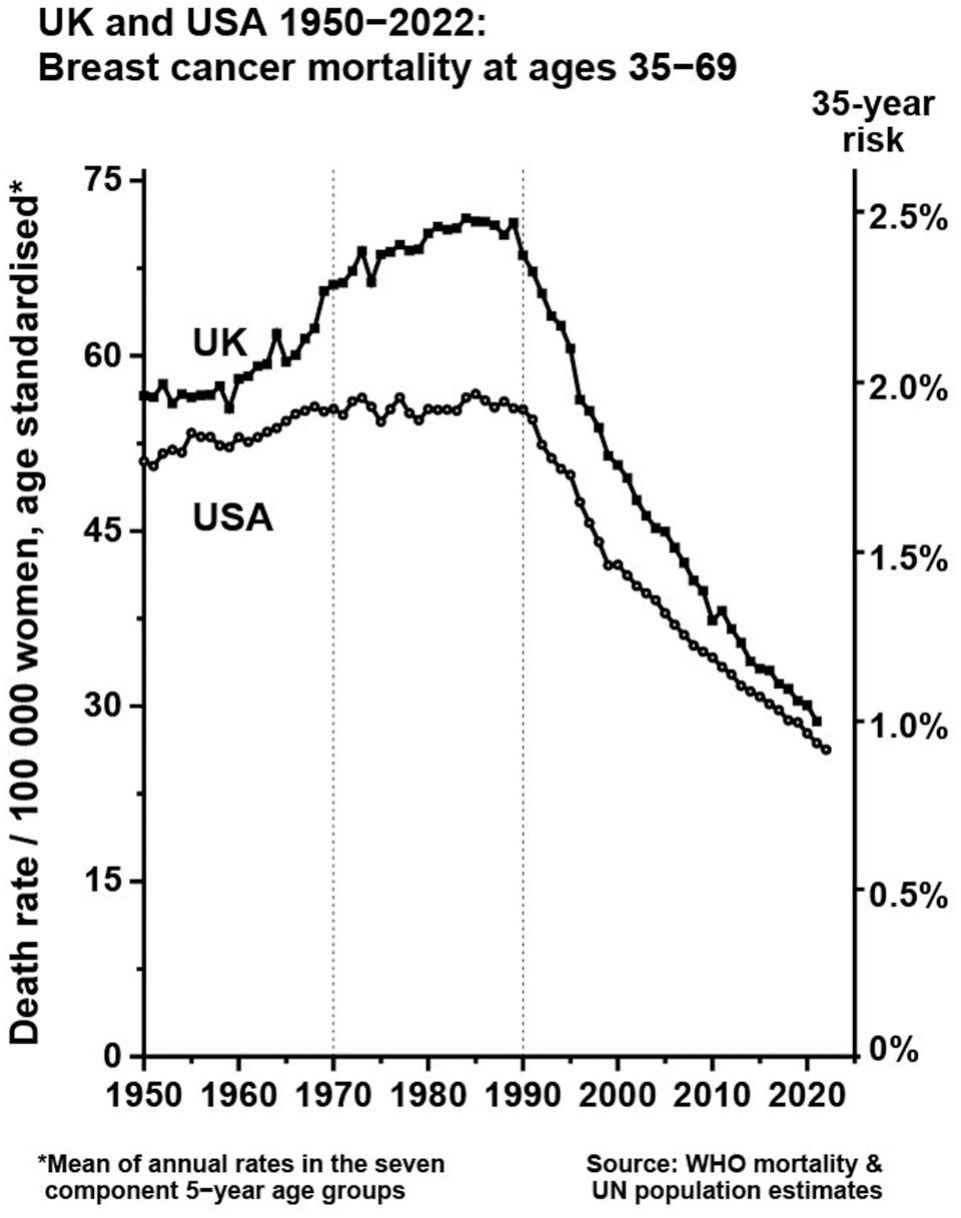
Fall in breast cancer mortality rates in the United Kingdom and United States in people aged 35–69 years (1950-2022). The age-standardized mortality rate is a mean of annual rates in the 7 component 5-year age groups (ages 35-39, 40-44, 45-49, 50-54, 55-59, 60-64, and 65-69 years). Data are from the WHO Mortality Database and UN World Population Prospects 2022 revision. Graph reproduced with permission from the Early Breast Cancer Trialists’ Collaborative Group (Hongchao Pan).

The breast cancer mortality rate in many countries in Europe and North America has fallen by 40% over 3 decades^6^ (Figure 2). This overall reduction in breast cancer mortality rate of roughly 2% per year is the combination of several moderate effects which we will discuss below.

## Early detection versus early diagnosis

The terms early detection and early diagnosis are often used interchangeably but have distinct meanings. Early detection refers to identifying cases among asymptomatic populations through screening, whilst early diagnosis is focused on providing comprehensive diagnostic and therapeutic care at the earliest possible stage to women with symptoms.

In less developed countries, late-stage presentation of women with breast cancer is common, ranging from 30%-50% to 75% in Latin America and Sub-Saharan Africa, respectively.^7^ The underlying reasons for delays in diagnosis are multifactorial and include educational and financial barriers coupled with a lack of high-quality healthcare provision.^8^

Population-based mammographic screening programmes started in Europe and North America in the late 1980s and have been shown to reduce breast cancer mortality by 20% among women who attend screening.^9^ Population-based screening programmes are labour and resource intensive and require robust quality assurance to ensure effectiveness and safety.

The average age of presentation of breast cancer is younger in less developed countries, and as such, the clinical and cost-effectiveness of population-based mammographic screening programmes is uncertain. Where there are resource constraints, emphasis should be placed on strategies to improve health literacy and ensuring the provision of high-quality accessible care for symptomatic presentation to facilitate the earlier diagnosis of breast cancer.^7^

## Treatment for breast cancer

Comprehensive breast cancer management comprises accurate diagnosis together with access to effective local (surgery and radiotherapy) and systemic therapies. The treatment recommendations for breast cancer are reliant on the accurate reporting of key tumour characteristics such as oestrogen and HER2 receptor status. In high-income countries, most patients have access to high-quality pathological services, but this figure is below one-third in low-income countries.^10^

It is estimated that only 25% of the world’s population has access to safe and timely cancer surgery, and this differs widely across resource settings with nearly 100% availability in more developed countries compared to just 27% in less developed countries.^11^ Similarly, only 29% of the least developed countries have operating radiotherapy services.^12^ The WHO essential medicine list, which includes anticancer treatments, aims to provide a comprehensive resource to guide the development of national formularies. However, there are well-described challenges in the procurement, accessibility, affordability, and safe delivery of cancer drugs which need to be addressed. For example, about 90% of the new anticancer drugs are consumed in the most developed countries, and the remaining 10% is accounted for by the rest of the world.^6^^,^^8^^,^^10^

## Initiatives to tackle the challenge

In response to the global challenge of breast cancer and observed disparities in outcomes between more and less developed countries various initiatives have been launched with the broad aims of improving survival from this treatable disease.

During 2002-2018, the Breast Health Global Initiative developed resource appropriate strategies to effective breast cancer care to take account of the unequal provision of healthcare and competing priorities in different resource settings.^13^

In 2021, the World Health Organisation launched the Global Breast Cancer Initiative (GBCI) to provide guidance to strengthen health systems with the aim of reducing global breast cancer mortality by an annual average of 2.5% which over a 20-year period would save 2.5 million lives. The initiative consists of 3 pillars including health promotion for early detection; timely diagnosis, and comprehensive breast cancer management.^6^

A further important consideration in countries with fragmented healthcare systems is that a cancer diagnosis in a household with limited resource can incur catastrophic healthcare expenditure. The need for universal healthcare coverage, meaning that all individuals can access quality healthcare services without incurring financial hardship is a key sustainable development goal. An increasing level of development in a country has been positively associated with national breast cancer care quality.^14^

## Impact of conflict on cancer care: Sudan as a case study

One of the main challenges in building and strengthening healthcare systems in low- and middle-income countries is economic and political instability.

Sudan is a country in Northeast Africa and has faced decades of civil wars and political unrest. The country is also facing a growing burden of cancer. In 2022, breast cancer was the most commonly diagnosed cancer and the leading cause of cancer-related mortality among women and the number of cases are expected to rise.^1^

Ongoing conflict has resulted in the displacement of over 12 million people and almost half of the population currently face high levels of food insecurity. The most recent conflict has led to the collapse of an already underfunded healthcare system, with less than a quarter of healthcare facilities still functioning in the worst affected areas.^15^

In 2021, Sudan had 4 cancer centres with a total of 10 radiotherapy machines to serve a population of approximately 45 million. The majority of patients were treated at the largest centre in the capital Khartoum.^16^ Previous efforts to decentralize cancer care services and establish provincial cancer centres to offer diagnostic services, limited surgical procedures, and basic chemotherapy have had limited impact on generating capacity.

Historically Sudan provided cancer care to refugee patients from neighbouring countries, many of which were affected by conflict and fragile healthcare systems. However, the collapse of the healthcare system, particularly in Khartoum, has led to significant disruption of cancer care services, leaving many patients without access to any care. Reported challenges include shortages in the workforce due to forced displacement and unpaid salaries, and in difficulties in accessing healthcare facilities due to unsafe travel routes and high transportation costs.^17^ Without immediate actions to restore the healthcare system and ensure the provision of necessary medications, a rise in cancer-related deaths in Sudan is inevitable.

## Summary

Breast cancer is a global health challenge of increasing importance. A significant reduction in mortality rates has been achieved over the last 3 decades in more developed countries largely due to early detection, timely diagnosis and improved access to effective treatments. However, disparities persist for women in less developed regions, resulting in avoidable deaths from a commonly curable disease. There remains an urgent need to address this challenge which will require a collective global effort.
